# Analysis of 2897 hospitalization events for patients with chronic kidney disease: results from CKD-JAC study

**DOI:** 10.1007/s10157-019-01730-9

**Published:** 2019-04-09

**Authors:** Satoshi Iimuro, Tetsuji Kaneko, Yasuo Ohashi, Tsuyoshi Watanabe, Kosaku Nitta, Tadao Akizawa, Seiichi Matsuo, Enyu Imai, Hirofumi Makino, Akira Hishida, for the CKD-JAC Investigators

**Affiliations:** 1 Teikyo Academic Research Center, Teikyo University, 2-11-1, Kaga, Itabashi-ku, Tokyo, Japan; 2 Department of Integrated Science and Engineering for Sustainable Society, Chuo University, Bunkyo-ku, Tokyo, Japan; 3 Japan Organization of Occupational Health and Safety Fukushima Rosai Hospital, Iwaki-city, Fukushima Japan; 4 Department of Nephrology, Tokyo Women’s Medical University, Shinjuku-ku, Tokyo, Japan; 5 Division of Nephrology, Department of Medicine, Showa University School of Medicine, Shinagawa-ku, Tokyo, Japan; 6 Department of Nephrology, Nagoya University, Nagoya, Aichi Japan; 7 Nakayamadera Imai Clinic, Takarazuka, Hyogo Japan; 8 Okayama University, Okayama, Okayama Japan; 9 Yaizu City Hospital, Yaizu, Shizuoka Japan

**Keywords:** Diabetes mellitus, Diabetic kidney disease, Cardiovascular disease, Pre-renal replacement therapy, Japanese patient survey 2008

## Abstract

**Background:**

Chronic kidney disease is a known risk factor for end-stage renal and cardiovascular diseases. However, data are limited on the causes of hospitalization in patients with chronic kidney disease of maintenance period. This study aimed to aggregate hospitalization data of CKD patients and to determine the high-risk population. In addition, we compared CKD population to general population.

**Methods:**

We conducted a post hoc analysis of the chronic kidney disease-Japan cohort study, a multicenter prospective cohort study of 2966 patients with chronic kidney disease with a median 3.9 years of follow-up. We examined the hospitalization reasons and analyzed the risk factors.

**Results:**

We found 2897 all-cause hospitalization events (252.3 events/1000 person-years), a hospitalization incidence 17.1-fold higher than that in an age- and sex-matched cohort from the general Japanese population. Kidney, eye and adnexa, and heart-related hospital admissions were the most common. All-cause hospitalization increased with chronic kidney disease stage and with the presence of diabetes. Patients with diabetes at enrollment had 345.7 hospitalization events/1000 person-years, which is considerably higher than 196.8 events/1000 person-years for those without diabetes. Survival analysis, using hospitalization as an event, showed earlier all-cause hospitalization with the progression of chronic kidney disease stage and diabetes. Cardiovascular disease hospitalizations were more strongly influenced by diabetes than chronic kidney disease stage.

**Conclusions:**

Patients with chronic kidney disease and diabetes are highly vulnerable to hospitalization for a variety of diseases. These descriptive data can be valuable in predicting the prognosis of patients with chronic kidney disease.

**Electronic supplementary material:**

The online version of this article (10.1007/s10157-019-01730-9) contains supplementary material, which is available to authorized users.

## Introduction

Chronic kidney disease (CKD) is a global public health problem [1]. Multiple cohort studies have been initiated worldwide to investigate its causes and effects [2]. The chronic renal insufficiency cohort (CRIC) study [3, 4] in the United States enrolled 4000 participants with CKD, and the resulting data have been used in many publications. On a strong cooperative relationship with the CRIC organizers, Japanese researchers initiated the chronic kidney disease-Japan cohort (CKD-JAC) study, a multicenter prospective cohort study of Asian patients with stages 3, 4, or 5 CKD living in Japan, aged 20–75 years that monitored patients for 4 years [5, 6]. Data from these studies are being used to determine disease-risk profiles for CKD patients and establish risk factors predicting CKD progression [7, 8].

The CRIC, CKD-JAC, and other cohort studies have clearly established CKD as an independent risk factor for end-stage kidney disease (ESKD), cardiovascular disease (CVD), and all-cause death [4, 7, 9]. Proteinuria, hypertension, diabetes, and dyslipidemia can worsen CKD [10–13], and diabetes is particularly important in countries where it is becoming increasingly common, such as Japan. Diabetic nephropathy is the primary cause of ESKD in Japan, and diabetes accounts for 45% of all ESKD cases. Chronic glomerulonephritis, which accounted for 60% of ESKD cases in Japan approximately 30 years ago, is now the etiology in only 20% of cases [14].

Hospitalization is another concern for CKD patients. Studies on hospitalizations are limited but report a relatively high incidence of hospitalization for arteriovenous shunting, CVD, and infection [15]. In another study, all-cause hospitalization increased with CKD stage [16]. Meanwhile, Japanese medical checkup data have shown that a lower estimated glomerular filtration rate (eGFR) is associated with increased risk of all-cause hospitalization and CVD death [17].

CKD patients are hospitalized not only for CKD, but for diabetes and related diseases. However, there are no reports on CKD patients’ hospitalization for other diseases or the contribution of CKD to them.

We aimed to determine the frequency and causes of hospitalization of CKD patients, to elucidate the prognosis using hospitalization as an indicator, and find high-risk population among them.

## Materials and methods

### Study design

CKD-JAC design has been published previously [5]. Inclusion criteria included the absence of polycystic kidney disease, HIV, liver cirrhosis, and cancer, and no history of receiving a transplant, or dialysis. The baseline disease, demographic data, and results of the primary analysis have been published previously [6–8]. The study protocol was approved by the institutional review boards at the institutions involved in the study, and CKD-JAC was conducted in accordance with the Declaration of Helsinki. For this study, formal approval and informed consent were not required as we analyzed previously published data. As for past history, diabetes was defined as HbA1C values of 6.5% or higher, taking antidiabetics and/or primary doctors’ reporting of with/without diabetes at enrollment. Diabetic nephropathy and glomerulonephritis were defined by primary doctors’ reporting regarding main cause for CKD at enrollment. HbA1c was expressed using NGSP.

### Hospitalization data

Hospitalizations were recorded from the enrollment date until the patient began dialysis. Thus, the data reflect only the maintenance period of CKD. The following hospitalization data were collected: (1) date of hospitalization and discharge, (2) diagnoses (maximum three), and (3) treatment (maximum three). Diagnosis and treatment could be entered using the code of dialysis outcomes and practice patterns study [18], but for many hospitalization events, the entries were free-form. For each hospitalization, the primary disease was identified by referring to the diagnosis and treatment. Hospitalizations were classified into 12 main disease groups: [01] infectious diseases, [02] malignant neoplasm, [03] endocrine, nutritional, and metabolic diseases, [04] diseases of the eye and adnexa, [05] diseases of the circulatory system, [06] heart diseases, [07] diseases of the respiratory system, [08] diseases of the digestive tract, [09] hepato-biliary-pancreatic diseases, [10] kidney diseases, [11] other 1 (otorhinolaryngology, dermatology, orthopedic conditions including bone and muscle, urology, obstetrics, and gynecology), and [12] other 2 (benign neoplasm, trauma, emergency, hematologic disease, psychiatric, and neurological). These groups are referred to as CKD-JAC classifications.

In addition to the CKD-JAC classification, diseases were also coded by the Japanese Ministry of Health, Labor and Welfare (MHLW) disease classification codes, based on the 2003 edition of the International Statistical Classification of Diseases and Related Health Problems [19]. Using disease classification codes enabled comparisons between CKD-JAC data and other epidemiological data.

Through these processes, for each hospitalization, one main disease name, and CKD-JAC and MHLW disease classification codes were identified. Relationships between the MHLW and CKD-JAC classification codes are shown in Supplementary Table 1.

### Control population

Hospitalization data for a general population comparable to the CKD-JAC population were obtained from the Patient Survey of 2008, published every 3 years by the Japanese MHLW [20], which provides random stratified sampling of patients who use medical care institutions across Japan. We used the 2008 edition because the CKD-JAC research began in 2006 and the next edition was likely affected by the major earthquake in northeast Japan in 2011. To compare the patient survey and CKD-JAC hospitalization data, we matched the patient survey population to the CKD-JAC population using sex, age, and person-years of observation as adjustment factors.

### Statistical analyses

The incidence of hospitalization was expressed as both the total number of hospitalization events and number of events per 1000 person-years. Kaplan–Meier survival analysis was applied for the time to first hospitalization, and survival curves were used to estimate the event-free survival rate. Even when patients had been hospitalized for other diseases, they were not removed from the group “at risk” for the first hospitalization event for ESKD or CVD. For this descriptive analysis of hospitalization, statistical significance was not assessed.

## Results

### Baseline demographics and disease-related data

From 3087 patients enrolled, 121 patients were excluded for some reasons, and finally, 2966 patients were observed for a median of 3.9 years [7, 8]. The mean age was 60.3 ± 11.6 years (38% women). CKD stages at enrollment were stage 3a [306 patients (10%)], stage 3b [1045 (35%)], stage 4 [1149 (39%)], stage 5 [466 (16%)] [7]. Diabetic patients were 1117 (62.3%), especially those with DM nephropathy were 612 (20.6%). Glomerulonephritis patients were 946 (31.9%). A total of 11,484.55 person-years were observed.

### Frequency of hospitalization for each disease

We observed 2897 all-cause hospitalization events (252.3 hospitalizations/1000 person-years, Table 1a). Supplemental Supplementary Table 1 shows specifics of each disease classification in the CKD-JAC classification. The most common reason for hospitalization was [10] kidney disease (86.1 events/1000 person-years, Supplementary Table 2). A total of 989 renal hospitalizations were observed mostly due to ESKD (*n* = 758), such as hospitalization to create a vascular access or start dialysis (Table 2). CKD stage was strongly associated with the number of ESKD hospitalizations: 4.9 events/1000 person-years for stage 3a CKD and 230.6 events/1000 person-years for stage 5 CKD.

**Table 1:** CKD-JAC classification for all hospitalizations (per 1000 patients)

CKD-JAC classification	All	Male	Female	Male/female
	Per 1000 person-years			
(a) All study subjects/subjects grouped by sex				
01. Infectious diseases	19.7	20.7	18.0	1.2
02. Malignant neoplasm	17.7	21.0	12.4	1.7
03. Endocrine, nutritional and metabolic diseases	16.6	16.3	17.1	1.0
04. Diseases of the eye and adnexa	15.0	14.9	15.1	1.0
05. Diseases of the circulatory system	12.7	15.2	8.8	1.7
06. Heart diseases	24.6	30.5	15.1	2.0
07. Diseases of the respiratory system	3.7	4.7	2.2	2.1
08. Diseases of digestive tract	15.7	15.3	16.2	0.9
09. Hepato-biliary-pancreatic diseases	6.4	7.2	4.9	1.5
10. Diseases of kidney	86.1	97.3	68.4	1.4
11. Others 1	22.8	18.2	30.1	0.6
12. Others 2	11.3	10.2	13.0	0.8
Total	252.3	271.7	221.4	1.2

CKD-JAC classification	CKD stage			
	3a	3b	4	5
	Per 1000 person-years			
(b) Subjects grouped by CKD stage				
01. Infectious diseases	18.9	17.7	22.1	18.7
02. Malignant neoplasm	13.2	22.5	17.6	9.0
03. Endocrine, nutritional, and metabolic diseases	19.7	13.4	18.3	18.1
04. Diseases of the eye and adnexa	14.8	14.4	16.7	12.0
05. Diseases of the circulatory system	9.9	12.2	15.6	8.4
06. Heart diseases	17.3	29.3	21.9	25.3
07. Diseases of the respiratory system	7.4	3.4	3.6	2.4
08. Diseases of the digestive tract	6.6	17.0	19.8	7.8
09. Hepato-biliary-pancreatic diseases	3.3	8.2	6.3	4.2
10. Kidney diseases	18.9	31.6	92.4	255.3
11. Other 1	26.3	23.5	23.7	16.3
12. Other 2	14.8	10.3	11.5	10.8
Total	171.0	203.6	269.4	388.4

CKD chronic kidney disease, CKD-JAC chronic kidney disease-Japan cohort

**Table 2:** Hospitalization for kidney diseases in patients grouped by CKD disease stage

		CKD stage				
		All	3a	3b	4	5
c-1402	Nephrotic syndrome	9 (0)	0 (0)	7 (0)	2 (0)	0 (0)
c-1403	Other glomerular diseases	47 (0)	11 (0)	26 (0)	8 (0)	2 (0)
c-1404	Renal tubulo-interstitial diseases	8 (0)	0 (0)	4 (0)	3 (0)	1 (0)
c-1405	Chronic renal failure	848 (758)	6 (6)	76 (63)	357 (304)	409 (380)
c-1406	Other renal failure	77 (5)	6 (0)	19 (0)	40 (2)	12 (3)
Total		989	23	132	410	424
Per 1000 person-years		86.1	18.9	31.6	92.4	255.3
Hospitalization not for ESKD		231	17	69	104	41
Per 1000 person-years		20.1	14.0	16.5	23.4	24.7
Hospitalization for ESKD		758	6	63	306	383
Per 1000 person-years		66.0	4.9	15.1	69.0	230.6

The CKD-JAC Kidney diseases classification [10] includes 989 events of the disease classification code a-1401 (glomerular disease, renal tubulo-interstitial disease, and renal failure) from the Ministry of Health, Labour and Welfare (MHLW) major classifications of disease. These specifics were expressed using the MHLW disease classification’s sub-classification code. The numbers within parentheses are the number of hospitalizations related to end-stage kidney disease

*CKD* chronic kidney disease, *ESKD* end-stage kidney disease, *CKD-JAC* chronic kidney disease-Japan cohort

The next most common reasons for hospitalization were CVD-related issues ([05] circulatory system and [06] heart), [01] infectious disease, and [02] malignant neoplasm, at 37.3, 19.7, and 17.7 events/1000 person-years, respectively. We observed 428 CVD-related hospitalization events: ischemic heart disease, 13.1 events/1000 person-years; stroke (cerebral infarction and other cerebrovascular diseases) 5.8 events/1000 person-years (Supplementary Table 2). The most common infection in group [01] was pneumonia. The most common type of [02] malignant neoplasm was gastrointestinal (Supplementary Table 3). [03] Endocrine, nutritional, and metabolic diseases accounted for 16.6 events/1000 person-years, with diabetes mellitus accounting for 9.3 events/1000 person-years.

### Sex differences in hospitalization rates on CKD-JAC

All-cause hospitalization was slightly higher in men (271.7 hospitalization events/1000 person-years) than women (221.4 events/1000 person-years). Hospitalizations for [07] respiratory system diseases and [06] heart were 2.1-fold and 2.0-fold more frequent in men, respectively. Hospitalization rates owing to [02] malignant neoplasm, [05] circulatory system, [09] hepato-biliary-pancreatic, and [10] kidney diseases were 1.7-fold, 1.7-fold, 1.5-fold, and 1.4-fold higher in men, respectively (Table 1a).

### Differences in hospitalization rates by CKD stage

**Fig. 1:**
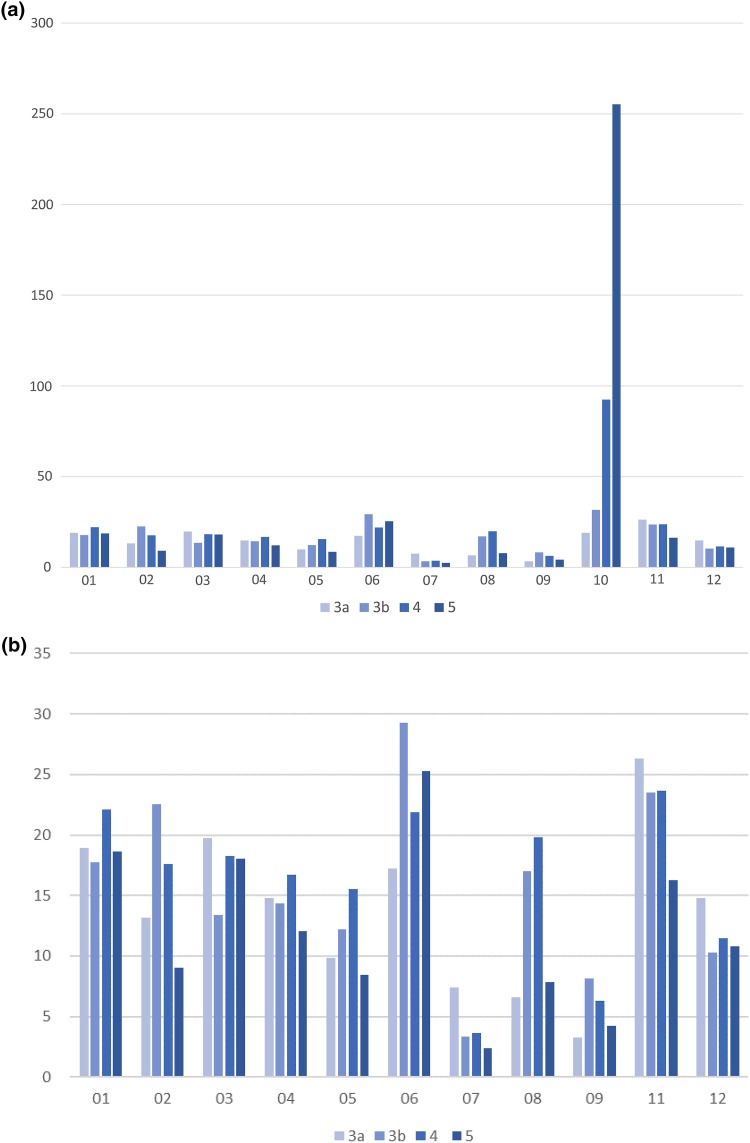
Hospitalization for each CKD-JAC classification by CKD stage. **a** Events per 1000 person-years with all classifications. **b** Events per 1000 person-years with [10] kidney diseases excluded [11]. Other one includes otorhinolaryngology, dermatology, orthopedic conditions including bone and muscle, urology, obstetrics, and gynecology [12]; other two include benign neoplasm, trauma, emergency, hematologic disease, psychiatric, and neurological. *CKD-JAC* chronic kidney disease-Japan cohort; *CKD* chronic kidney disease, *3a* CKD stage 3a, *3b* CKD stage 3b, *4* CKD stage 4, *5* CKD stage 5

**Fig. 2:**
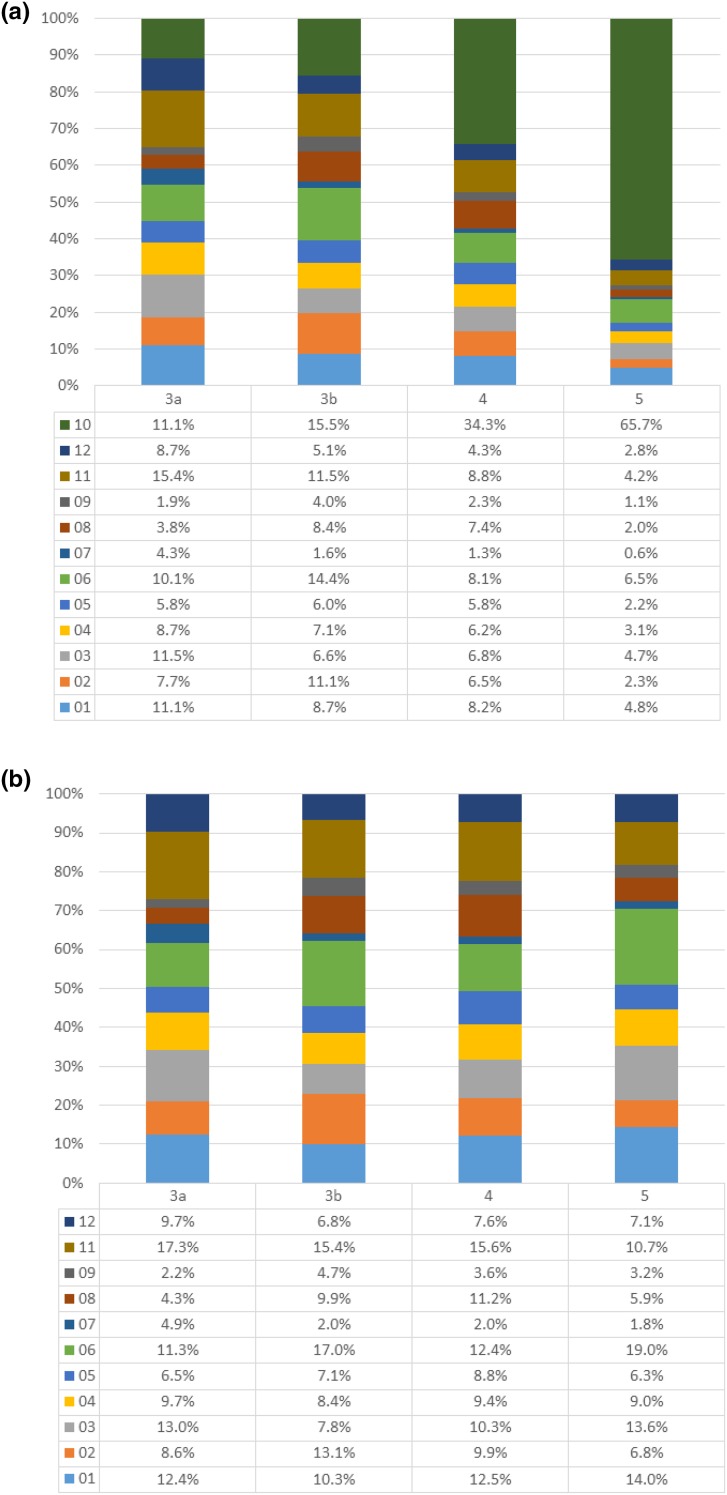
The percentage of hospitalizations attributable to each disease classification. **a** Patients at different CKD stages with all classifications. **b** Patients at different CKD stages with [10] kidney diseases excluded. *CKD* Chronic kidney disease, *3a* CKD stage 3a, *3b* CKD stage 3b, *4* CKD stage 4, *5* CKD stage 5

All-cause hospitalization increased with CKD stage; stage 3a had 171.0 events/1000 person-years, increasing to 388.4 events/1000 person-years at stage 5. Hospital admissions for [10] kidney disease increased sharply at stage 5 (Fig. 1a). For [05] circulatory system, [06] heart, and [08] digestive tract, hospitalizations increased from stage 3a to 4 and decreased in stage 5 (Table 1b). For most other diseases, the number of hospitalizations was consistent across CKD stages (Fig. 1). Patients were hospitalized for a variety of diseases at CKD stages 3a and 3b, but at stages 4 and 5, there was a sharp increase in the percentage of patients hospitalized for [10] kidney diseases (Fig. 2a).

### Effects of underlying disease

All-cause hospitalization was higher in diabetic patients than in those who were diabetes-free at enrollment (345.7 events/1000 person-years vs. 196.8 events/1000 person-years; Table 3). Within the subgroup of patients with diabetic nephropathy, total hospitalizations reached 381.2 events/1000 person-years. The classifications most affected by diabetes were [03] endocrine, nutritional, and metabolic, [06] heart [04] eye and adnexa, and [10] kidney. Patients who got CKD mainly from glomerulonephritis could keep relatively good conditions rather than not only those with diabetes but also those with other underlying diseases (175.2 events/1000 person-years; Table 3).

**Table 3:** Hospitalization events for subjects grouped by underlying disease status

CKD-JAC classification	DM(−)	DM (+)	DM(−)		DM (+)	
			Others	Glomerulo-nephritis	DM nephropathy (−)	DM nephropathy (+)
	Per 1000 person-years					
01. Infectious diseases	17.6	23.1	21.5	14.0	27.3	19.5
02. Malignant neoplasm	16.7	19.4	21.2	12.4	21.8	17.4
03. Endocrine, nutritional and metabolic diseases	6.7	33.4	8.6	4.8	18.7	46.0
04. Diseases of the eye and adnexa	8.6	25.7	8.3	8.9	18.7	31.7
05. Diseases of the circulatory system	11.0	15.7	14.9	7.3	13.2	17.8
06. Heart diseases	12.5	44.9	17.5	7.8	34.4	53.8
07. Diseases of the respiratory system	2.8	5.4	4.0	1.6	5.6	5.2
08. Diseases of digestive tract	15.7	15.7	22.1	9.7	18.7	13.0
09. Hepato-Biliary-Pancreatic diseases	5.6	7.7	5.4	5.7	10.6	5.2
10. Diseases of kidney	69.8	113.6	64.5	74.8	91.1	132.9
11. Others 1	19.7	28.0	20.9	18.6	32.9	23.9
12. Others 2	10.3	13.1	10.9	9.7	11.1	14.8
Total	196.8	345.7	219.8	175.2	304.2	381.2

DM (−) and DM (+) at enrollment, and DM (−)/glomerulonephritis, DM (−)/other, DM (+)/diabetic nephropathy (−) and DM (+)/diabetic nephropathy (+) at enrollment

*DM* diabetes mellitus; *CKD-JAC* chronic kidney disease-Japan cohort

When we aggregated the hospitalizations without [10] kidney disease (mainly for ESKD), the diabetes group had nearly twofold more hospitalizations than the non-diabetes group (232.1 events/1000 person-years vs. 127.0 events/1000 person-years). The diabetes group had higher hospitalization rates for [03] endocrine, nutritional, and metabolic and [04] eye and adnexa (mostly diabetic retinopathy). Even when we adjusted for those classifications, diabetic patients had more hospitalizations, even for conditions unrelated to diabetes (172.9 vs. 111.7 hospitalization events). The greatest difference between the two groups was for [06] heart disease. However, even without that classification, there were 128.1 hospitalization events/1000 person-years in the diabetes group and 99.2 in the non-diabetes group.

### Hospitalization duration

Median durations of each disease categories were from about 12 days to 14 days. There were little differences between male and female, CKD stages and four categories of underlying diseases (Supplementary Fig. 1).

### Comparison to a control population

The control population from the patient survey of 2008 [20] had 14.8 hospitalization events/1000 person-years (Table 4a), higher in men (16.8 events/1000 person-years) than women (11.4 events/1000 person-years), of which tendency was also observed in CKD-JAC.

**Table 4:** Disease classifications for hospitalization in the control population

CKD-JAC classification	Control			CKD-JAC	CKD-JAC/control
	All	Male	Female		
(a) Data on the control cohort and comparison between the CKD-JAC and control populations					
01. Infectious diseases	0.7	0.8	0.4	19.7	29.1
02. Malignant neoplasm	2.2	2.7	1.3	17.7	8.1
03. Endocrine, nutritional, and metabolic diseases	0.4	0.5	0.3	16.6	39.9
04. Diseases of the eye and adnexa	0.1	0.1	0.2	15.0	100.5
05. Diseases of the circulatory system	2.0	2.5	1.3	12.7	6.3
06. Heart diseases	0.5	0.7	0.3	24.6	46.3
07. Diseases of the respiratory system	0.3	0.4	0.1	3.7	13.6
08. Diseases of the digestive tract	0.5	0.6	0.3	15.7	32.0
09. Hepato-biliary-pancreatic diseases	0.3	0.4	0.2	6.4	18.6
10. Kidney diseases	0.4	0.5	0.3	86.1	218.0
11. Other 1	1.7	1.6	1.9	22.8	13.5
12. Other 2	5.6	6.1	4.5	11.3	2.0
Total	14.8	16.8	11.4	252.3	17.1

CKD-JAC classification	Female			Male		
	CKD-JAC	Control	JAC/ctrl	CKD-JAC	Control	JAC/ ctrl
(b) Comparisons between female and male subjects in the CKD-JAC and control populations						
01. Infectious diseases	18.0	0.4	42.2	20.7	0.8	25.0
02. Malignant neoplasm	12.4	1.3	9.5	21.0	2.7	7.8
03. Endocrine, nutritional, and metabolic diseases	17.1	0.3	49.7	16.3	0.5	35.4
04. Diseases of the eye and adnexa	15.1	0.2	99.0	14.9	0.1	101.4
05. Diseases of the circulatory system	8.8	1.3	6.7	15.2	2.5	6.2
06. Heart diseases	15.1	0.3	50.0	30.5	0.7	45.6
07. Diseases of the respiratory system	2.2	0.1	15.8	4.7	0.4	13.2
08. Diseases of the digestive tract	16.2	0.3	49.1	15.3	0.6	26.1
09. Hepato-biliary-pancreatic diseases	4.9	0.2	20.4	7.2	0.4	17.9
10. Kidney diseases	68.4	0.3	243.6	97.3	0.5	209.3
11. Other 1	30.1	1.9	15.8	18.2	1.6	11.6
12. Other 2	13.0	4.5	2.9	10.2	6.1	1.7
Total	221.4	11.4	19.4	271.7	16.8	16.1

With data from the Ministry of Health, Labour and Welfare Patient Survey of 2008 and age, sex, and observation time as adjustment factors, a control cohort from the general population was created to match the CKD-JAC group

*CKD-JAC* Chronic kidney disease-Japan Cohort,* ctrl* control

The CKD-JAC population had 17.1-fold more all-cause hospitalization events than controls. Hospitalizations were markedly higher in the CKD-JAC population for all individual conditions, especially the [10] kidney (218.0-fold) and [04] eye and adnexa (100.5-fold) classifications. However, even when we removed the classifications closely related to CKD ([10] kidney, [03] endocrine, nutritional and metabolic, and [04] eye and adnexa) from the all-cause hospitalization data, hospitalizations were still 9.7-fold more frequent in the CKD-JAC population than in controls. This trend was relatively consistent in both men and women (Table 4b).

### Time to the first hospitalization based on CKD stage

#### All-cause hospitalization

**Fig. 3:**
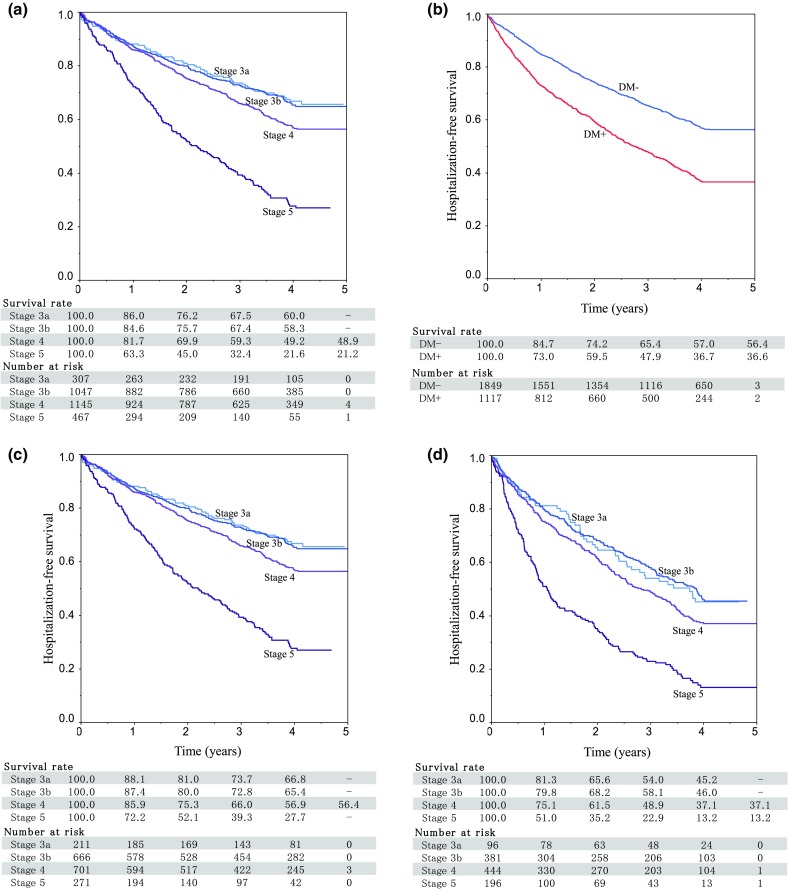
Kaplan–Meier survival curves for the first hospitalization due to any cause. **a** CKD stage. **b** DM status. **c** CKD stage and DM (−). **d** CKD stage and DM (+) [11]. Other one includes otorhinolaryngology, dermatology, orthopedic conditions including bone and muscle, urology, obstetrics, and gynecology [12]; other two include benign neoplasm, trauma, emergency, hematologic disease, psychiatric, and neurological. *CKD* chronic kidney disease, *DM* diabetes mellitus

Survival curves were similar for CKD stages 3a and 3b. For stage 4, the number of hospitalizations began to rise after the first year. The stage 5 survival curve diverged immediately. During the first year of follow-up, only 63% of stage 5 patients were still not hospitalized, compared to 86% of stage 3a patients, a difference of 23%. By the fourth year, that difference had increased to 39% [22% (stage 5) vs. 60% (stage 3a); Fig. 3a].

Diabetes profoundly affected hospitalization-free survival (Fig. 3b). In patients grouped by CKD stage and diabetes mellitus (DM + or DM−), hospitalization occurred earliest in stage 5 DM + patients, followed by stage 5 DM−, next stage 4 DM+, and then stage 3b DM + or stage 3a DM+. First-year hospitalization-free rates for these groups were 51%, 72%, 75%, 80%, and 81%, respectively. The time to the first hospitalization was longest for stage 3a DM − patients, stage 3b DM − patients, and stage 4 DM − patients (Fig. 3c, d).

#### Hospitalization for CVD

**Fig. 4:**
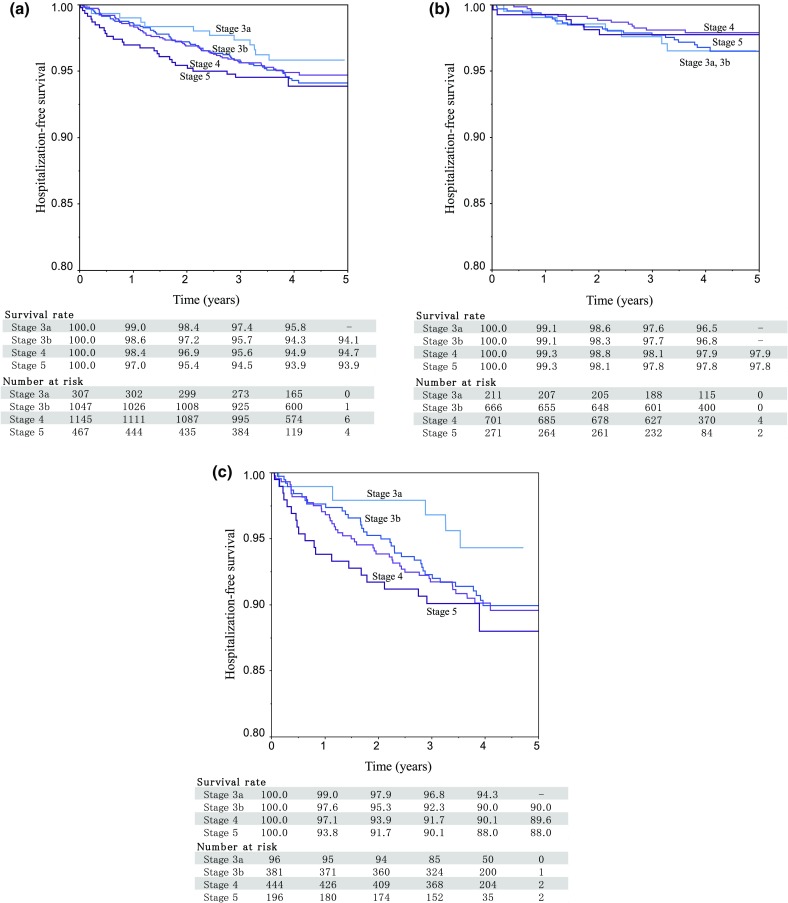
Kaplan–Meier survival curves for the first hospitalization due to CVD. **a** CKD stage. **b** CKD stage and DM (−). **c** CKD stage and DM (+). Events included ischemic heart diseases and cerebral infarction from [06] heart diseases and [05] diseases of the circulatory system [11]. Other one includes otorhinolaryngology, dermatology, orthopedic conditions including bone and muscle, urology, obstetrics, and gynecology [12]; other two include benign neoplasm, trauma, emergency, hematologic disease, psychiatric, and neurological. *CKD* chronic kidney disease, *CVD* cardiovascular disease, *DM* diabetes mellitus

Survival analysis was applied to analyze the time to first hospitalization for sub-classifications [a-0902] ischemic heart diseases, [a-0903] other heart diseases, [a-0904] cerebral infarction, and [a-0905] other cerebrovascular diseases in CVD-related issues ([05] circulatory system). After 4 years, hospitalization rates remained low at each stage (Fig. 4a). When we grouped the patients by CKD stage and DM status, diabetes was a clear risk factor for CVD-related hospitalization (Fig. 4b, c).

## Discussion

In this study, we used data from nearly 4 years of follow-up on 2966 participants to profile risk factors and underlying causes of hospitalization in CKD patients. With 2897 hospitalization events (252.3 events/1000 person-years), we have strong data supporting a few important findings in this vulnerable patients.

These data demonstrate that CKD patients are at high risk of hospitalization for many diseases. All-cause hospitalization was 17.1-fold more common in CKD-JAC patients than in the control cohort. CKD is a risk factor for ESKD and CVD, and the high hospitalization rate for the [10] kidney diseases (86.1 events/1000 person-years) and [06] heart (24.6 events/1000 person-years) classifications is consistent with previous findings [7, 8, 15]. However, the magnitude of that effect in this study was greater than expected: a 218.0-fold higher risk for [10] kidney diseases and 46.3-fold higher risk for [06] heart diseases than in the control cohort. Hospitalizations were also more frequent for diseases of the [04] eye and adnexa (100.5-fold) and [03] endocrine, nutritional, and metabolic diseases (39.9-fold). Both classifications are related to diabetes, which is a major underlying disease in CKD. Even after we removed [04] eye and adnexa [03] endocrine, nutritional, and metabolic, as well as [10] kidney diseases, in which ESKD plays a major role, CKD-JAC hospitalizations were still substantially more frequent (9.7-fold) than hospitalizations in controls. This indicates that CKD is a risk factor for many diseases, and CKD patients are highly vulnerable to many conditions that may require hospitalization. This analysis provides a unique comprehensive profile of all kinds of hospitalizations.

Our study also provides valuable data on the effects of CKD stage on hospitalization. Hospitalizations for [10] kidney disease increased dramatically with CKD stage, and hospitalizations for [05] circulatory system and [06] heart diseases were greater at CKD stage 4 than at stage 3a (Fig. 1b). Previous research has clearly shown that CKD stages 3 through 5 are independent predictors of ESKD and CVD [16, 21–24]. Our data confirm these findings, except for decreased hospitalizations at stage 5 for [05] circulatory system and [06] heart diseases. That may be because only the main disease names from hospitalization data including maximum three diagnoses were analyzed. Competing risk analysis will be done in the future.

We were able to investigate potential risk of underlying diseases on hospitalization. All-cause hospitalization was approximately 1.8 times higher in diabetic patients (345.7 events/1000 person-years in the DM + group vs. 196.8 in the DM − group). Among those with diabetes, hospitalizations were even more frequent in the subgroup of patients diagnosed with diabetic nephropathy (381.2 events/1000 person-years). Results were similar even after excluding disease classifications closely related to diabetes. Clearly, diabetes affects hospitalization for many diseases, both related and unrelated to diabetes. In the group previously diagnosed with glomerulonephritis, the number of hospitalization events was low for most diseases except those in [10] kidney. These findings demonstrate that the disease underlying CKD has important prognostic value and the subpopulation of patients with diabetic nephropathy is at particularly high risk.

Survival analysis also illustrated the major impact of diabetes on CKD. The time to first hospitalization was shorter in patients with a higher CKD stage and in those with diabetes. If we evaluate all-cause hospitalizations, the time to first hospitalization was shorter in patients with CKD stage 3b and diabetes than those with CKD stage 4 without diabetes. For CVD-related issues, diabetes had an unexpectedly strong effect on the survival time. The time to CVD-related hospitalization was shorter in diabetic patients than in patients who were diabetes-free, across all CKD stages.

This study had some limitations. First, at CKD stage 5, we expected an increase in hospitalizations related to renal diseases. These patients may have been hospitalized for other diseases, but the diagnoses could be obscured by the primary disease name. To address this, we plan a future investigation of competing risks. Second, there is a problem to be considered whether CKD-JAC data had an appropriate representativeness of CKD patients in Japan. Our data were from large leading hospitals that have nephrologists in residence, but the control data were obtained from a survey of hospitals of all sizes, not necessarily with nephrologists in residence. The results of this comparison may reflect not only differences in the incidence of hospitalization between CKD patients and the general population but also differences between large nephrology hospitals and the general population. At large hospitals, patients have the advantage of receiving treatment optimized to their disease state. Therefore, the incidence of hospitalization may be lower than that in ordinary hospitals. Third, proper evaluation of CKD during maintenance period must be based on the comparison between CKD-JAC group and general population which meets inclusion and exclusion criteria of CKD-JAC. However, it was impossible to do that, so we used patient survey data as the substitute. Even though the patient survey population was matched to the CKD-JAC population using sex, age, and person-years of observation as adjustment factors, these data contain both patients who get hospitalized at a certain day and those who have been hospitalized on the same day. That means we may overestimate the incidence of hospitalization in general population. In addition, more and more patients over 75 years old have started dialysis in recent years [25]. These aged people have higher risk for hospitalization than younger generation under 75 years old, not only for ESKD but also various aging conditions. To evaluate the risk of CKD more precisely, these elderly patients should have included to our study. Further study is expected for evaluating CKD on elderly people.

These might result in underestimation of the overall extent of hospitalization of CKD patients. However, we believe that the magnitude of differences described in this paper is sufficient to justify the generalizability of our results.

As for hospitalization durations, they are likely to be affected by the medical assurance system in Japan, so other suitable studies to evaluate them will be needed.

In addition to validating the previously observed increased risk for CVD and ESKD, this study clearly shows the extreme vulnerability of CKD patients to many other diseases. These data are highly valuable for predicting prognoses in CKD patients, in addition, for identifying high-risk population among CKD patients.

## Electronic supplementary material

Below is the link to the electronic supplementary material.

Supplementary material 1 (DOCX 532 KB)

Supplementary material 2 (XLSX 55 KB)

Supplementary material 3 (XLSX 27 KB)

Supplementary material 4 (XLSX 29 KB)

Supplementary material 5 (XLSX 20 KB)

Supplementary material 6 (DOCX 21 KB)
