# Supplementary material for: Analysis of 2897 hospitalization events for patients with chronic kidney disease: results from CKD-JAC study
Source: Clin Exp Nephrol. 2019 Apr 9;23(7):956–68. doi: 10.1007/s10157-019-01730-9 (PMC6555784; doi:10.1007/s10157-019-01730-9)
Supplement: Supplementary file 1 — Supplementary material 1 (DOCX 532 KB) [file 10157_2019_1730_MOESM1_ESM.docx]

Analysis of 2897 hospitalization events for patients with chronic kidney disease: Results from CKD-JAC study, *Clinical and Experimental Nephrology*, Iimuro et al.

**Supplementary figure 1a - d shows distributions of admission durations.**

1. admission durations of each disease by CKD-JAC classification
2. sex
3. CKD stages
4. four categories of underlying diseases for CKD
5. **CKD-JAC classification**

[11] Other 1 includes otorhinolaryngology, dermatology, orthopedic conditions including bone and muscle, urology, obstetrics, and gynecology; [12] Other 2 includes benign neoplasm, trauma, emergency, hematologic disease, psychiatric, and neurological

1. **Female/Male**

1. **CKD stage**

1. **Four categories of underlying diseases for CKD**
