# Supplementary material for: Analysis of 2897 hospitalization events for patients with chronic kidney disease: results from CKD-JAC study
Source: Clin Exp Nephrol. 2019 Apr 9;23(7):956–68. doi: 10.1007/s10157-019-01730-9 (PMC6555784; doi:10.1007/s10157-019-01730-9)
Supplement: Supplementary file 6 — Supplementary material 6 (DOCX 21 KB) [file 10157_2019_1730_MOESM6_ESM.docx]

Analysis of 2897 hospitalization events for patients with chronic kidney disease: Results from CKD-JAC study, *Clinical and Experimental Nephrology*, Iimuro et al.

**Supplementary Table 3 Hospitalizations for Malignant Neoplasm**

| **Malignant neoplasm of digestive organs** | **67** |
| --- | --- |
| c-0202，Esophagus | 1 |
| c-0203，Stomach | 13 |
| c-0204，Colon | 10 |
| c-0205，Recto sigmoid junction and rectum | 11 |
| c-0207，Liver and intra-hepatic bile ducts | 12 |
| c-0208，Gallbladder and other and unspecified parts of biliary tract | 4 |
| c-0209，Pancreas | 8 |
| c-0210，Other digestive organs | 8 |
| **Malignant neoplasm of respiratory and intra-thoracic organs** | **7** |
| c-0211，Larynx | 1 |
| c-0212，Trachea, bronchus, and lung | 5 |
| c-0213，Other sites in the respiratory system and intra-thoracic organs | 1 |
| **Malignant neoplasm of urinary tract** | **12** |
| c-0226，Kidney | 7 |
| c-0227，Bladder | 5 |
| **Malignant neoplasm of breast and female genital organs** | **12** |
| c-0218，Breast | 10 |
| c-0223，Female genital organs | 2 |
| **Malignant neoplasm of male genital organs** | **17** |
| c-0224， Prostate | 17 |
| **Others** | **88** |
| c-0214，Bone and articular cartilage | 1 |
| c-0217，Mesothelial and soft tissue | 1 |
| c-0230，Central nervous system | 2 |
| c-0231，Thyroid gland | 3 |
| c-0233，Non-Hodgkin's lymphoma | 1 |
| c-0235，Immunoproliferative diseases, multiple myeloma, and malignant plasma cell neoplasms | 8 |
| c-0236，Unable to be classified | 72 |
| **Total** | **203** |

CKD-JAC, Chronic Kidney Disease-Japan Cohort

The CKD-JAC malignant neoplasm category [02] includes 203 hospitalizations for cancer. The specifics were expressed using the Ministry of Health, Labour and Welfare disease classification’s sub-classification code, broadly classified by organs.
